# *Bacillus subtilis* Protects the Ducks from Oxidative Stress Induced by *Escherichia coli*: Efficacy and Molecular Mechanism

**DOI:** 10.3390/antiox11101951

**Published:** 2022-09-29

**Authors:** Chong Li, Yang Li, Shuzhen Li, Si Chen, Guohua Liu, Xuejuan Deng, Wenhuan Chang, Huiyi Cai

**Affiliations:** 1 Key Laboratory for Feed Biotechnology of the Ministry of Agriculture and Rural Affairs, Institute of Feed Research, Chinese Academy of Agriculture Sciences, Beijing 100081, China; 2 Precision Livestock and Nutrition Laboratory, Teaching and Research Centre (TERRA), Gembloux Agro-Bio Tech, University of Liège, 5030 Gembloux, Belgium; 3 Department of Molecular Cell Biology, Samsung Medical Center, Sungkyunkwan University School of Medicine, Suwon 16419, Korea; 4 National Engineering Research Center of Biological Feed, Beijing 100081, China

**Keywords:** *Bacillus subtilis*, pekin duck, *Escherichia coli*, oxidative stress, ribosome, oxidative phosphorylation

## Abstract

*Bacillus subtilis* has been widely used in animal husbandry as a potential alternative to antibiotics due to its excellent bacteriostasis and antioxidant activity. This study aims to investigate the effects of *Bacillus subtilis* on the protection of ducks from *Escherichia coli* infection and its mechanism. The four experimental groups include the negative control group, positive control group, antibiotic group and *Bacillus subtilis* group. Ducks in positive, antibiotic and *Bacillus subtilis* groups are orally administered with *Escherichia coli* and equivalent saline solution for the negative group. The results show that supplements with *Bacillus subtilis* enhances the performance and health status of the infected ducks. Moreover, *Bacillus subtilis* alleviates the increase in globulin, LPS and MDA, and the decrease in albumin, T-AOC and T-SOD in the serum caused by *Escherichia coli* infection. *Bacillus subtilis* also attenuates injury in the intestine and partially reverses the increase in ROS production and the depletion of ATP in the jejunum. These effects are accompanied with the change of related genes of the ribosome (13.54%) and oxidative phosphorylation (6.68%). Collectively, *Bacillus subtilis* alleviates the damage caused by *Escherichia coli* infection in ducks by activating ribosome and oxidative phosphorylation signaling to regulate antioxidant and energy metabolism.

## 1. Introduction

Oxidative stress caused by an imbalance between the excessive production of free radicals and the antioxidant defense is an important component of biological damage and also the main source of serious diseases [1]. It was reported that the endotoxin produced by pathogenic *Escherichia coli* (*E. coli*), such as lipopolysaccharide (LPS), can cause excessive production of reactive oxygen or nitrogen species, which exceeds the defense capacity of the host and increases the level of oxidative stress products such as MDA, ultimately causing a series of oxidative injuries [2,3]. What is more, severe oxidative stress caused by *E. coli* infection leads to impairment of energy metabolism through interference with oxidative phosphorylation pathways, ultimately compromising host antioxidant capacity, immune response and productive performance [1].

Ducks and chickens are the main victims of avian pathogenic *E. coli*. The infected individuals show progressive debilitation, which results in damage to organs throughout the body and eventual death due to functional failure [4,5]. Antibacterial drugs and vaccines are the main treatments for *E. coli* infections; however, the multidrug-resistant and complicated pathogenicity of *E. coli* continues to increase and attracts endless attention [6].

The challenges outlined above have prompted a global search for alternatives to antibiotics. Probiotics are widely used in humans and have gained acceptance as an animal feed additive to reduce the use of antibiotics gradually [7,8]. In the poultry industry, probiotics have been shown to stimulate innate immunity and overall health by preventing pathogen infections, thereby improving growth performance, promoting intestinal morphology and other functions [9,10,11,12]. *Bacillus subtilis* (*B. subtilis*) is a kind of probiotic that produces subtilisin, polymyxin, nystatin, short bacitracin and other active substances. It has been widely used in animal husbandry as a potential alternative to antibiotics due to its excellent bacteriostasis, antioxidant, immune and growth improvement functions [13,14,15]. However, *B. subtilis* has been less studied in ducklings, especially the ducklings exposed to *E. coli*. In recent years, we have been concerned about the negative effect of pathogenic *E. coli* on waterfowl and have successfully established a model of *E. coli* infection in Pekin ducklings [16]. Based on previous work, we compared the effects of *B. subtilis* L6 and virginiamycin on growth performance, antioxidation function and the intestinal health of ducks challenged with *E. coli* in the current study. In addition to the physiological and biochemical analyses, the RNA-Seq was also used to determine possible molecular mechanisms by which *B. subtilis* relieved intestinal oxidative stress caused by *E. coli* infection in Pekin ducklings.

## 2. Materials and Methods

### 2.1. Preparation of E. coli O88 and B. subtilis L6

The strain of pathogenic *E. coli* O88 was obtained from China Veterinary Culture Collection Center (CVCC). The probiotic *B. subtilis* L6 in microcapsules (viable count ≥1.0 × 10^10^ CFU/g, powder state) was provided by Challenge Biotechnology Co., LTD (Beijing, China). The viable *B. subtilis* count in feed was determined based on Nikoskelainen (2003) [17].

### 2.2. Experimental Design

The experiment was conducted in Nankou pilot base of the Chinese Academy of Agricultural Sciences. The methods for animal experiments were set out by the National Institute of Animal Health and research reporting follows the guidelines of ARRIVE [18]. A total of 192 newly hatched, male lean Pekin ducklings were randomly allocated into 4 treatment groups with 6 replicates of 8 ducks each replicate. The 4 treatment groups were negative control group (NC), positive control group (PC), 30 mg/kg virginiamycin group (ANT) and 2.5 × 10^9^ CFU/kg *B. subtilis* L6 group (BS), respectively. The basal diets meet the nutritional requirements of the ducks as determined by the National Research Council (NRC, 1994) and the *Nutrient Requirements of Meat-type Duck* published by the Ministry of Agriculture of the People’s Republic of China, NY/T 2122-2012 (Supplementary Table S1). The experiment lasted for 28 days.

### 2.3. Oral Challenge

**Figure 1:**
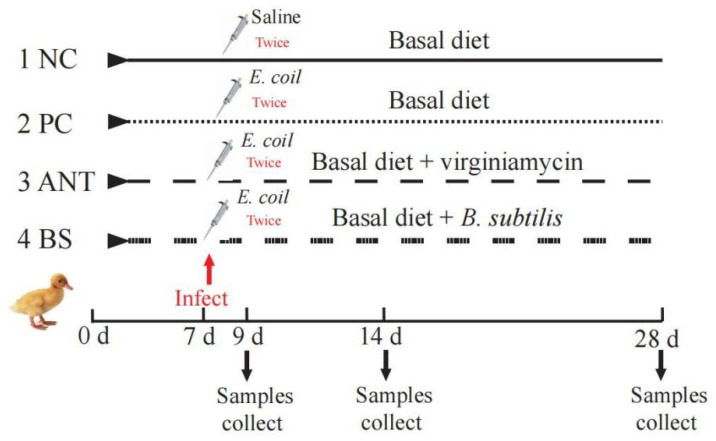
Workflow for the experiment. NC–negative group, basal diet without *E. coli* challenge. PC–positive group, basal diet with *E. coli* challenge. ANT–antibiotic group, basal diet + virginiamycin (30 mg/kg) with *E. coli* challenge. BS–basal diet + 2.5 × 10^9^ CFU/kg *B. subtilis* with *E. coli* challenge.

The infection model was established based on our previous protocol [16]. Briefly, the frozen *E. coli* O88 was thawed and cultured in Luria-Bertani (LB) broth to activate three times (37 °C, 12 h). Bacteria were resuspended in sterilized 0.9% saline solution and counted by plate cultivation. On day 7, the ducks in PC, ANT and BS groups were orally administered with 0.2 mL *E. coli* (3 × 10^9^ CFU/mL) twice, 8 h apart and equivalent volumes of 0.9% sterile saline solution for the NC group. The workflow is shown in Figure 1.

### 2.4. Sampling

On days 9, 14 and 28, after fasting 6 h, all ducks were weighed and the feed intake was measured on a per cage basis. Average daily feed intake (ADFI), average daily gain (ADG) and the feed intake/weight gain (F/G) ratio were calculated. After a 6 h fast, one duck (close to the average BW) from each replicate was selected and euthanized by electric stunning, and then the blood samples (2.5 mL) were taken from the wing vein into an anticoagulant-free vacuum test tube (5 mL), centrifuged at 3000× *g* for 10 min and stored at −20 °C. The ducks were opened longitudinally, both ceca were ligated and aseptically removed from the gastrointestinal tract for cecal *E. coli* analysis. The middle portion of jejunum (1.5 cm) was cut off and flushed residual digesta with ice-cold phosphate-buffered saline (PBS), and then fixed in 10% neutral formalin for intestinal histomorphology analysis [19]. The mucosa of jejunum segments was gently scraped and snap-frozen in liquid nitrogen and then transferred to a −80 °C freezer till analyzed. 

### 2.5. Cecal E. coli

Cecal contents were obtained from the ligated ceca on days 9, 14 and 28. The viable counts of *E. coli* were analyzed by the method of Manafi [20]. Briefly, the cecal contents of each bird were pooled and serially diluted. *E. coli* was counted on Eosin Methylene Blue agar plates after incubation 24 h at 37 °C. The colony-forming unit was defined as distinct colony at least one mm in diameter. 

### 2.6. Serum Indices

The serum albumin, total protein and LPS levels were measured using commercial assay kits (Nanjing Jiancheng Bioengineering Institute, Nanjing, China. The catalog numbers were A028-1-1, A045-3-2 and H255, respectively) by colorimetric method (UV-2550, Shimadzu, Japan). Because the total protein in serum mainly consists of globulin and albumin, the globulin content was obtained by subtracting the albumin value from that of the total protein [21]. Serum total antioxidant capacity (T-AOC), activity of total superoxide dismutase (T-SOD) and malonaldehyde (MDA) concentration were determined by the commercial assay kits (Nanjing Jiancheng Bioengineering Institute, Nanjing, China. The catalog numbers were A015-2-1, A001-1-2 and A003-2-2, respectively) with an automated fluorescence instrument (MultiskanM™ SkyHigh, Thermo Fisher Scientific, Waltham, MA, USA).

### 2.7. Intestinal Morphology

The middle portion of jejunum was fixed in formalin for 24 h, embedded in paraffin, deparaffinized, dehydrated and stained, observed the jejunum morphological structure by Motic Panthera Moticam 5 trinocular microscope BA210LED (Motic Incorporation Ltd., Hong Kong, China) and analyzed by Moticam digital imaging system (Motic images Software Plus 2.0, Motic Incorporation Ltd., Hong Kong, China). Villus height and crypt depth were measured at least 10 well-oriented villus and then calculated the villus height/crypt depth ratio (V/C) ratio. The measurement has referred to the method of Lin [22]. 

### 2.8. ROS Production and ATP Level of Jejunum

The reactive oxygen species (ROS) production of jejunum was measured by using the ROS commercial assay kits (S0033S. Beyotime Institute of Biotechnology, Haimen, China) and 2,7-dichlorofluorescein diacetate as fluorescence probe. The specific process was carried out as the method of Zhang [23,24]. The adenosine triphosphate (ATP) level was determined by the commercial ATP assay kits (BC0305. Solarbio, Beijing, China).

### 2.9. RNA-Seq Analysis of the Jejunum

The RNA-Seq analysis was performed on 12 jejunum samples from PC and BS groups. The middle portion of jejunum was collected on day 14. Total RNA in jejunum tissues was extracted by Trizol kits (Invitrogen, Carlsbad, CA, USA) and treated by DNase I to avoid DNA contamination. RNA concentration was determined by Nanodrop 2000. Total RNA was analyzed qualitatively and quantitatively using Agilent 2100 (Agilent Technologies, Inc., Palo Alto, CA, USA). RNA-seq libraries were generated using NEBNext Ultra RNA Library Prep Kit for Illumina (NEB, Ipswich, MA, USA). Libraries were sequenced on a HiSeq2500 platform (Illumina Inc., San Diego, CA, USA) to generate Paired-end 100 bp raw reads.

The adaptor sequences and low-quality sequence reads were removed from the data sets using fastx-toolkit tool. These clean reads were then mapped to the reference genome sequence (Anas_platyrhynchos: ASM874695v1). Gene expression levels were quantified as fragments per kilo base of transcript per million fragments (FPKM) mapped from different jejunum samples [25]. Differential expression analysis was performed using the DESeq2 R package (1.6.3) [26]. DEGs with |fold change (FC)| ≥ 2 and false discovery rate (FDR) < 0.05 were considered as differentially expression. The differentially expressed genes (DEGs) were annotated and enriched by Kyoto Encyclopedia of Genes and Genomes (KEGG, http://www.kegg.jp/kegg/pathway.html, accessed on 13 September 2022) using the clusterProfiler R package (3.10.1).

### 2.10. RT-qPCR

Nine DEGs associated with “ribosome” and “oxidative phosphorylation” pathways, including Ribosomal protein lateral stalk subunit P2 (*RPLP2*), Mitochondrial ribosomal protein L23 (*MRPL23*), NHP2 ribonucleoprotein (*NHP2*), NADH-ubiquinone oxidoreductase core subunit V1 (*NDUFV1*), Cytochrome c oxidase subunit 7B mitochondrial (*COX7B*), ATP synthase membrane subunit F (*ATP5MF*), Ribosomal protein L22 like 1 (*RPL22L1*), ATPase H^+^ transporting V1 subunit A (*ATP6V1A*), NADH-ubiquinone oxidoreductase subunit A6 (*NDUFA6*) were selected and using RT-qPCR to confirm the accuracy and reliability of RNA-Seq results. The total RNA from the jejunum tissues was isolated by TRI-zol reagent (TIANGEN, Beijing, China) and reversely transcribed into complementary DNA (cDNA) pursuant. The concentration of total RNA was determined by a spectrophotometer (Ultrospec 2100 pro, GE Healthcare, Chicago, IL, USA) and purified by agarose gel electrophoresis. Then 500 ng total RNA was reversely transcribed into cDNA using the primescript of Fast Quant RT Kit (TIANGEN, Beijing, China). The qPCR was conducted by the Biosystems Bio-Rad Real-Time PCR system (Bio-Rad, Carlsbad, CA, USA) with the Brilliant SYBR Green qPCR Master Mix (Stratagene, La Jolla, CA, USA). The primers used are listed in (Supplementary Table S2). The beta-actin (*β-actin*) was used to normalize the expression of the targeted genes. The mRNA level of the relative genes was calculated using the method of 2^−ΔΔCt^ [27]. All samples were analyzed in triplicate and the geometric mean of internal references.

### 2.11. Statistical Analysis

Each replicate cage was considered as the experimental unit for growth performance data, whereas the individual duck in each replicate was the experimental unit for other parameters analysis (*n* = 6). The data were analyzed by a one-factor ANOVA procedure of SPSS19.0 software package for Windows (SPSS Inc., Chicago, IL, USA). Significant differences between groups were separated using Duncan’s multiple range test. Differences were considered significant at *p* < 0.05. The graphs were designed using GraphPad Prism 9 Project (GraphPad Software Inc., San Diego, CA, USA) and Origin 8.5 (Origin Lab, Berkeley, CA, USA).

## 3. Results

### 3.1. Oral Challenge, Growth Performance and Serum Parameters

**Figure 2:**
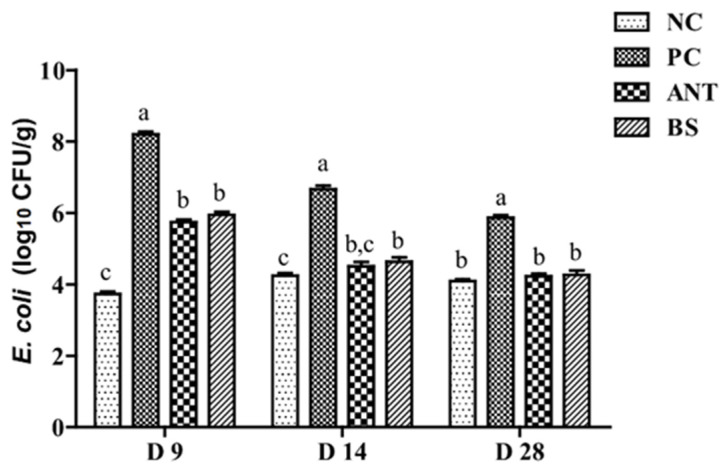
Effect of *B. subtilis* on the quantity of *E. coli* in the cecum of ducks challenged with *E. coli*. NC–negative group, basal diet without *E. coli* challenge. PC–positive group, basal diet with *E. coli* challenge. ANT–antibiotic group, basal diet + virginiamycin (30 mg/kg) with *E. coli* challenge. BS–basal diet + 2.5 × 10^9^ CFU/kg *B. subtilis* with *E. coli* challenge. Data are indicated as means ± SEM. ^a,b,c^ Values, for the same day, with different superscripts are significantly different (*p* < 0.05). The individual duck in each replicate was the experimental unit (*n* = 6).

The challenge with *E. coli* significantly increased the population of *E. coli* in the cecum of ducks on day 9 (*p* < 0.01, Figure 2), which indicates that the infection model was successfully established. When these birds were also treated with ANT or BS, the population of *E. coli* in their cecum showed a significant reduction when compared with the PC group on day 14 (*p* < 0.01). Moreover, the amount of *E. coli* was not a significant difference was found on day 28 between the ANT, BS and NC groups.

The response to growth performance is shown in Table 1. Ducks in the PC group had the lowest BW on day 14 and ADG during days 9–14 and had the highest F/G ratio during days 14–28 and overall (*p* < 0.05). *B. subtilis* supplementation significantly increased the BW and ADG during the whole period and decreased the F/G ratio during days 14–28 compared to the PC group (*p* < 0.05). There was no significant difference in ADG, ADFI and F/G ratio between the BS and ANT groups.

As shown in Table 2, the *E. coli* challenge significantly decreased the albumin level, A/G ratio and increased the LPS level on days 9 and 14, increasing the globulin level on day 9 (*p* < 0.05). While *B. subtilis* supplementation significantly increased the A/G ratio on day 14 compared to the PC group (*p* < 0.05). There was no significant difference in albumin, globulin and A/G ration between BS and ANT groups. 

**Figure 3:**
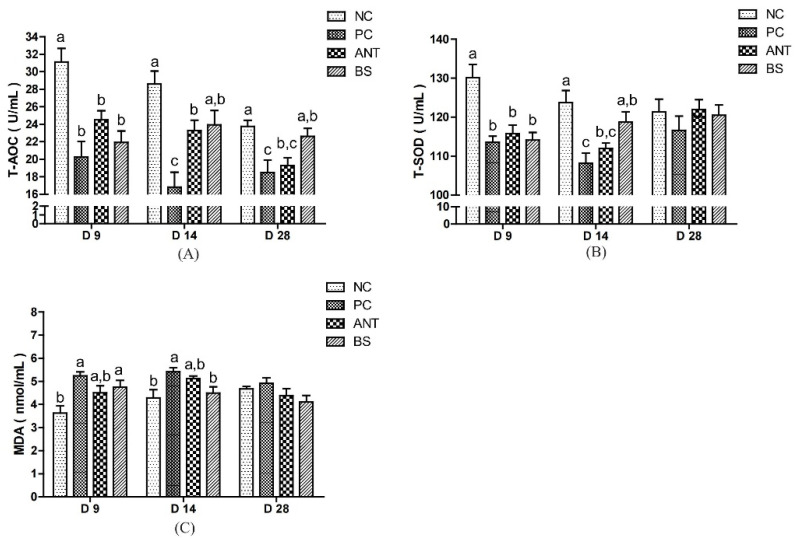
Effect of *B. subtilis* on serum antioxidant parameters of ducks challenged with *E. coli*. NC–negative group, basal diet without *E. coli* challenge. PC–positive group, basal diet with *E. coli* challenge. ANT–antibiotic group, basal diet + virginiamycin (30 mg/kg) with *E. coli* challenge. BS–basal diet + 2.5 × 10^9^ CFU/kg *B. subtilis* with *E. coli* challenge. Data are indicated as means ± SEM. The individual duck in each replicate was the experimental unit (*n* = 6). (**A**) T-AOC, total antioxidant capacity. (**B**) T-SOD, total superoxide dismutase. (**C**) MDA, malonaldehyde. ^a,b,c^ Values, for the same parameter and day, with different superscripts are significantly different (*p* < 0.05).

Serum antioxidant indices of ducks are shown in Figure 3. Compared with the NC group, the PC group had lowered the T-AOC level in the whole test stage, lower T-SOD and higher MDA level on days 9 and 14 (*p* < 0.05). The BS group had higher T-AOC levels on days 14 and 28, higher T-SOD levels on day 14 and lower MDA levels on day 14 than the PC group (*p* < 0.05). There was no difference in T-AOC, T-SOD and MDA levels between the BS and ANT groups. 

### 3.2. Intestinal Morphology, ROS Production and ATP Level

**Figure 4:**
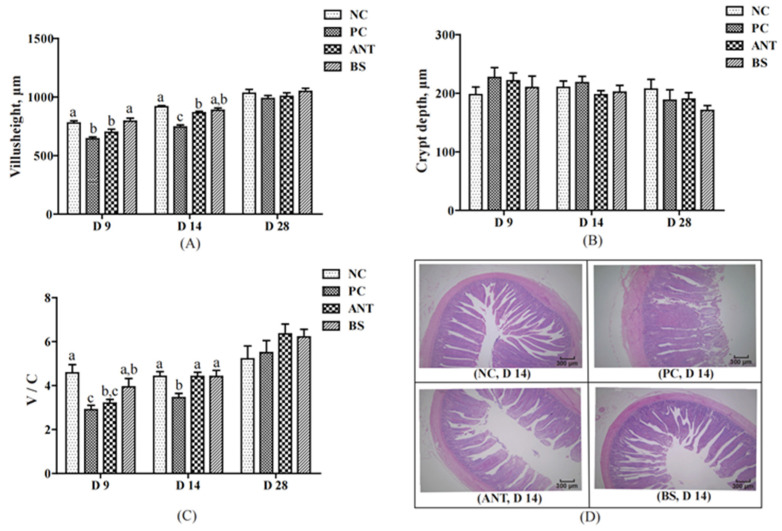
Effect of *B. subtilis* on jejunum histomorphology of ducks challenged with *E. coli*. NC–negative group, basal diet without *E. coli* challenge. PC–positive group, basal diet with *E. coli* challenge. ANT–antibiotic group, basal diet + virginiamycin (30 mg/kg) with *E. coli* challenge. BS–basal diet + 2.5 × 10^9^ CFU/kg *B. subtilis* with *E. coli* challenge. Data are indicated as means ± SEM. The individual duck in each replicate was the experimental unit (*n* = 6). (**A**) The villus height in jejunal of ducks. (**B**) The crypt depth in jejunal of ducks. (**C**) The ratio of villus height to crypt depth in jejunal of ducks. (**D**) Histological damage in jejunal of ducks on day 14. ^a,b,c^ Values, for the same parameter and day, with different superscripts are significantly different (*p* < 0.05).

Changes in the intestinal morphology of ducks are shown in Figure 4. Compared with the NC group, the villus height and V/C ratio of the PC group were lower on days 9 and 14 (*p*  <  0.05). The BS group had higher villus height and V/C ratio than the PC group on days 9 and 14 (*p*  <  0.05). The villus height was higher for ducks in the BS group than in the ANT group on day 9 (*p*  <  0.05). There was no significant difference in crypt depth among these four groups. The histological examination showed normal jejunal villous structure for the NC group, disorganized and interrupted and incomplete for the PC group, and to some extent repaired for the BS group.

**Figure 5:**
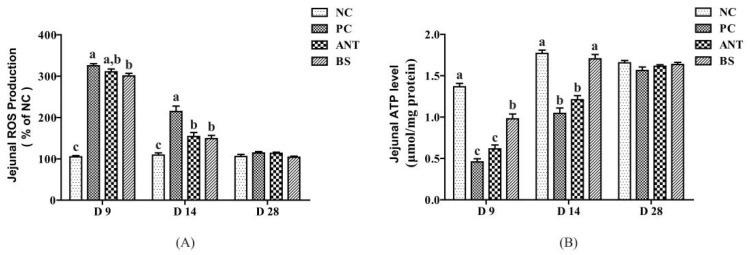
Effect of *B. subtilis* on the ROS production and ATP levels of jejunum in ducks challenged with *E. coli*. NC–negative group, basal diet without *E. coli* challenge. PC–positive group, basal diet with *E. coli* challenge. ANT–antibiotic group, basal diet + virginiamycin (30 mg/kg) with *E. coli* challenge. BS–basal diet + 2.5 × 10^9^ CFU/kg *B. subtilis* with *E. coli* challenge. Data are indicated as means ± SEM. The individual duck in each replicate was the experimental unit (*n* = 6). (**A**) ROS, reactive oxygen species. (**B**) ATP, adenosine triphosphate. ^a,b,c^ Values, for the same parameter and day, with different superscripts are significantly different (*p* < 0.05).

As shown in Figure 5, *E.coli* infection significantly increased the ROS production of jejunum on days 9 and 14 than the NC group (*p*  <  0.05). The antibiotic supplementation significantly decreased the ROS production on day 14 than the PC group (*p*  <  0.05). *B. subtilis* treatment significantly reduced ROS production on days 9 and 14 than the PC group (*p*  <  0.05). There was no significant difference in ROS production between the BS and the ANT groups. *E.coli* infection significantly decreased the ATP level of jejunum on days 9 and 14 than NC group (*p* < 0.05). While the *B. subtilis* significantly increased the ATP level than the PC and the ANT groups (*p* < 0.05). There was no significant difference between PC and ANT groups on days 9 and 14.

### 3.3. RNA-Seq Analysis of the Jejunum

To define the mechanism underlying the biological effects of *B. subtilis* that facilitates the recovery from *E. coli*-induced damage, the 12 sequencing libraries from the PC group (PC-1, PC-2, PC-3, PC-4, PC-5, PC-6) and the BS group (BS-1, BS-2, BS-3, BS-4, BS-5, BS-6) were constructed. After quality control of sequencing data, 20,587,116–31,570,825 clean reads were obtained to establish 12 RNA-Seq libraries. The clean data of each sample reached 6.14 GB, the GC content ranged from 50.45% to 53.05% and the percentage of Q30 base was above 94.32%. Sequence alignment of each sample was conducted against the designated reference genome with an efficiency from 87.89 to 91.80%. Statistics of the sequencing data are provided in Table 3.

**Figure 6:**
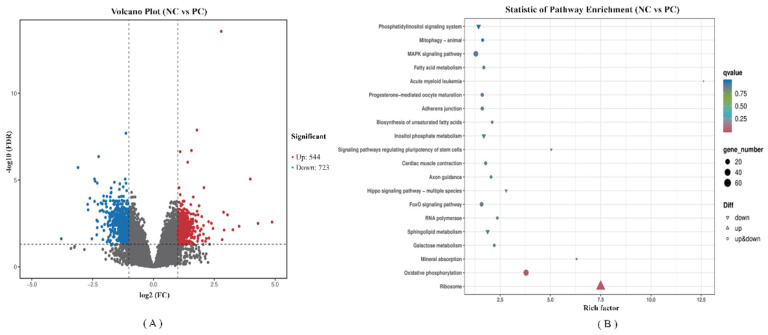
RNA-seq analysis of the jejunum tissue of ducks (PC VS BS). PC–positive group, basal diet with *E. coli* challenge. BS–basal diet + 2.5 × 10^9^ CFU/kg *B. subtilis* with *E. coli* challenge. (**A**) Volcano plot of differentially expressed genes. X-axis shows log2 (FC) of DEGs between each two groups, Y-axis represents the statistically significant negative logarithm value of the gene expression change; Each point in the volcanic map represents a gene, Red represents increased expression while green represents decreased expression; (**B**) Dot plot of KEGG pathway enrichment analyses of differentially expressed genes.

FPKM was applied to determine the expression of genes. Volcano plots of transcriptome sequencing data were used to visualize the distribution of DEGs between the two groups. A total of 1267 genes were differentially expressed between the PC and BS groups. Of those, 544 were up-regulated and 723 were down-regulated (Figure 6A).

We performed a KEGG pathway enrichment analysis for the 1267 DEGs, and a scatter plot of KEGG data was created by selecting the top 20 enriched pathways (Figure 6B). Among them, ribosome (ko03010) and oxidative phosphorylation (ko00190) were the most significant predictors (*p* < 0.01). All DEGs involved in the ribosome pathway were up-regulated, while DEGs involved in the oxidative phosphorylation pathway showed varied expression patterns. Taking a further step, the KEGG annotations of DEGs were classified according to the type of cellular processes, environmental information processing, genetic information processing, human disease, metabolism and organismal system pathways. A detailed classification is shown in Supplementary Figure S1. The 50 canonical pathways were observed among DEGs, of which the highest enriched pathways are ribosome (13.54%) and oxidative phosphorylation (6.68%). The top 10 key DEGs known functions related to these pathways are listed in Table 4 and Table 5, respectively. Compared with the PC group, *B. subtilis* supplementation increased the expression of ribosomal protein lateral stalk subunit, mitochondrial ribosomal protein and NHP2 ribonucleoprotein. *B. subtilis* also increased the expression of genes associated with oxidative phosphorylation metabolism, including NADH-ubiquinone oxidoreductase core subunit, mitochondrial Cytochrome c oxidase subunit and ATP synthase membrane subunit. These findings indicate that *B. subtilis* may be involved in the generation of ribosomes and energy metabolism.

### 3.4. Validation of Gene Expression by Using RT-qPCR 

**Figure 7:**
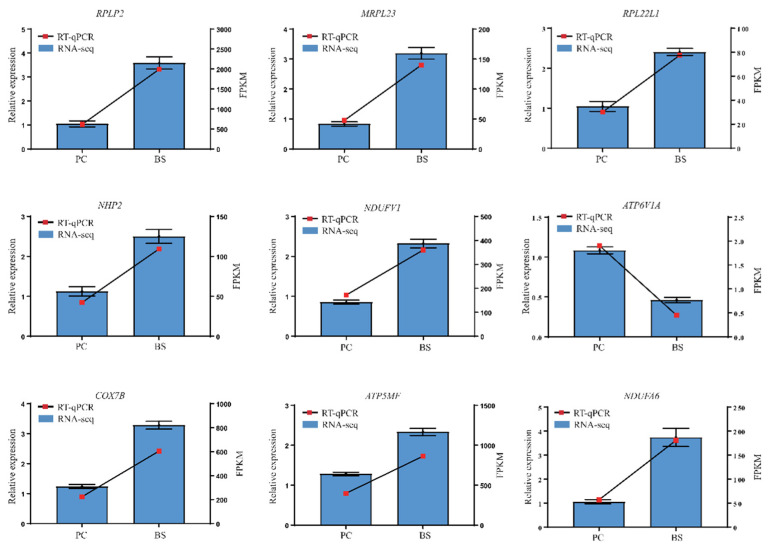
Relative expression levels from RT-qPCR. PC–positive group, basal diet with *E. coli* challenge. BS–basal diet + 2.5 × 10^9^ CFU/kg *B. subtilis* with *E. coli* challenge. The relative expression values were normalized to the *β*-actin gene. *RPLP2*: Ribosomal protein lateral stalk subunit P2; *MRPL23*: Mitochondrial ribosomal protein L23; *NHP2*: NHP2 ribonucleoprotein; *NDUFV1*: NADH-ubiquinone oxidoreductase core subunit V1; *COX7B*: Cytochrome c oxidase subunit 7Bmitochondrial; *ATP5MF*: ATP synthase membrane subunit F; *RPL22L1*: Ribosomal protein L22 like 1; *ATP6V1A*: ATPase H+ transporting V1 subunit A; *NDUFA6*: NADH-ubiquinone oxidoreductase subunit A6.

Nine genes involved in pathways of ribosomal *(RPLP2*, *MRPL23*, *NHP2*, *RPL22L1*) and oxidative phosphorylation (*NDUFV1*, *COX7B*, *ATP5MF*, *ATP6V1A*, *NDUFA6*) were selected for validation by RT-qPCR. Their results showed that, compared with PC ducks, *B. subtilis* increased the expression of *RPLP2*, *MRPL23*, *NHP2*, *RPL22L1*, *NDUFV1*, *COX7B*, *ATP5MF*, *NDUFA6* and decreased the expression of *ATP6V1A* to varying degrees. The changes in the relative expression levels of the RT-qPCR showed similar trends to the transcriptome-sequencing analyses (Figure 7), suggesting that the RNA-Seq data are reliable.

## 4. Discussion

Colibacillosis is the most common infectious bacterial disease of poultry that may cause host health damage and loss of production. Antibiotics are effective in the treatment of avian colibacillosis due to the large-scale use of antibiotic growth promoters (AGP), which has caused drug residues and resistance, and threatened food safety. *B. subtilis* is a kind of probiotic and a potential alternative to AGP, which can improve growth performance and gut health and even resist pathogenic bacteria. Therefore, the current study was designed to investigate the effects of the probiotic *B. subtilis* L6 on duck health compared with AGP. Our results showed that *E. coli* O88 decreased the body weight of ducks, but the supplementation of virginiamycin and *B. subtilis* improved the growth performance of ducks challenged with *E. coli.*, and there was no significant difference between them. This is consistent with previous studies that *E.coli* infection reduces productivity by threatening host health [16], but *B. subtilis* or its fermented products could alleviate the adverse effects on the growth performance of broilers challenged with LPS or *E. coli* [28,29]. 

The pathogenic *E. coli* could cause septic multiple organ inflammation and injury. Serum biochemical indexes are closely related to metabolism, in which ALB and GLB are the two major components of serum proteins. The albumin level represents the systemic inflammatory response and appears to be used as biomarkers of kidney or liver damage. The globulin level is associated with chronic inflammation and participates in the process of inflammation response [30]. The A/G ratio reflects the immune response capacity of the body, and the decrease in the ratio indicates the presence of immunologic injury [31]. LPS is the main outer component of *E. coli* and is also an important virulence factor for *E. coli* infection, which can cause oxidativee stress and immune response, seriously threatening the immune system of the host [32,33,34]. In the current study, *B. subtilis* alleviates the negative impacts of *E. coli.* In support of these findings, Manafi and Erinle reported that probiotics could play an active role in the regulation of serum biochemical parameters through multiple mechanisms when broilers were challenged with *E. coli* [20,33]. As the gram-negative bacterial cell wall component [35], LPS may leak into the bloodstream through the damaged intestinal mucosa [36], providing opportunities for the invasion of harmful metabolites and pathogens, consequently, triggering a robust systemic inflammatory response [37]. We found that pathogenic *E.coli* increased the levels of LPS in the serum, but this negative impact was well alleviated by *B. subtilis*, which suggested that specific probiotic strains could decrease LPS and maintain immune homeostasis.

The oxidative stress is caused by an imbalance between the antioxidant and pro-oxidative systems [38]. The T-SOD, T-AOC activity and MDA level are the main indices to evaluate the oxidation state of the body. Of these, T-SOD can regulate the balance in vivo through enzymatic reactions [39]. The T-AOC reflects the ability of the non-enzymatic antioxidant defense system [40]. MDA is an end product of lipid peroxidation and also a biomarker of oxidative stress [41]. It was reported that the pathogenic microorganism’s infection or LPS induced oxidative stress [42], while probiotics may enhance antioxidant defenses in tissues [43]. In the present study, the decrease in T-AOC and T-SOD activities and the increase in MDA concentration of the PC group indicated that the *E. coli* infection caused oxidative stress reactions in ducks. Conversely, *B. subtilis* alleviated the changes in the biomarker of oxidative stress, which might be due to the fact that *B. subtilis* counteracted oxidative stresses by promoting the early innate immune system [44]. In terms of the mechanism explanation, it is possible that *B. subtilis* competes with *E. coli* for attachment sites and nutrients in the intestine and reduces the initialization step of oxidative stress caused by *E. coli*. Moreover, *B. Subtilis* may also play some role in the repair of oxidative lesions [45,46].

The integrity of the gut structure is related to gut health and nutrient absorption [47]. Intestinal villus height, crypt depth and V/C ratio are important indicators to measure the intestinal barrier function and absorption ability [48]. *E. coli* infection could seriously damage the morphology and structure of the intestinal tract by decreasing the villi height and increasing the crypt depth [49]. Studies have shown that *B. subtilis* strains can improve gut histomorphological indices and promote the repair of tissue damage in animals infected by pathogenic bacteria [29,50]. Our results showed that *E. coli* infection severely disrupted the morphological structure of the jejunum in early-stage ducks; however, *B. subtilis* was more beneficial to maintaining the morphology of the intestinal epithelium and repairing intestinal barrier damage caused by *E. coli* infection than the virginiamycin, which also provided a reasonable explanation for the improvement in growth performance of ducks in the BS group.

Mitochondria are the main source of physiological and pathological ROS and also an important part of ROS elimination. The outbreak and excessive accumulation of ROS in the mitochondrial respiratory chain, which exceeds the scavenging capacity of the antioxidant system, may cause mitochondrial dysfunction, including impaired mitochondrial respiratory complex activity and reduced ATP level [51]. The current study indicated that ROS content in jejunum tissue increased, and ATP level decreased after the *E. coli* challenge, indicating that the mitochondrial respiratory chain is damaged.

In recent years, the effects of cellular energy metabolism in regulating intestinal inflammatory diseases have received increasing attention [52,53]. The mitochondrion plays a central role in energy metabolism homeostasis through the respiratory chain. ATP synthesis takes place in the mitochondria by oxidative phosphorylation [54,55]. Mitochondrial oxidative phosphorylation contributes to producing enough ATP for the body to use [56], and once this process is disrupted and ATP synthesis is also affected [57]. Inflammation and metabolic diseases are associated with mitochondrial dysfunction. It has been demonstrated that *E. coli* infection can damage the intestinal barrier and trigger mucosal inflammatory responses subsequently in ducks, which is an energy-consuming process [58]. In the previous study, ducks infected with *E. coli* impaired the growth performance of ducks through an interfering energy metabolism pathway, down-regulating gene expression related to the ribosome and oxidative phosphorylation [59]. In the present study, *B. subtilis* reduced intestinal oxidative stress through interfering with ribosome and energy metabolism by up-regulating the expression of genes that encode mitochondrial ribosome proteins, ribosome biogenesis and translation factors. There was evidence that increased expression of ribosomal protein genes was associated with increased cellular growth and that it could be regulated by a variety of upstream factors [60]. The potential mechanism may involve the lysate or secreted chemical inducer of probiotics [61]. Apart from this, *B. subtilis* up-regulated the oxidative phosphorylation complex genes and restored mitochondrial function damaged by *E. coli* infection. This was similar to the report that after the damage to the intestine, the activities of jejunal mitochondrial respiratory complexes and the level of ATP were reduced [22]. The mechanism was also demonstrated at the cellular level. The LPS-activated-macrophages are characterized by high glycolysis and low oxidative phosphorylation [62,63], *B. subtilis* may be reversed this process.

## 5. Conclusions

In conclusion, pathogenic *E. coli* O88 could cause systemic injuries to ducks, including oxidative stress, triggered inflammatory responses and impaired growth performance of ducks. These changes may be effectively mitigated by *B. subtilis* supplementation, and the effectiveness of the treatment was comparable to that of virginiamycin. The possible molecular mechanism was that *B. subtilis* impacted the energy metabolism of intestinal cells by up-regulating the pathways related to the ribosome and oxidative phosphorylation so as to reduce the oxidative stress and inflammatory response induced by *E. coli* O88 and promote growth performance.

## Figures and Tables

**Table 1:** Effect of *B. subtilis* on growth performance of ducks challenged with *E. coli*.

Parameter	Days	Dietary Treatment				SEM	*p*-Value
		NC	PC	ANT	BS		
BW, g	9	301.7 ^ab^	290.1 ^b^	311.0 ^a^	312.3 ^a^	2.746	0.006
	14	600.5 ^a^	557.5 ^b^	616.4 ^a^	614.8 ^a^	7.274	0.005
	28	1690.1 ^ab^	1558.1 ^b^	1740.2 ^a^	1787.9 ^a^	28.26	0.019
ADG, g/(duck-d)	1–9	27.6 ^ab^	27.1 ^b^	29.1 ^ab^	29.2 ^a^	0.331	0.036
	9–14	59.8 ^a^	52.8 ^b^	60.1 ^a^	60.2 ^a^	1.063	0.021
	14–28	77.5 ^ab^	72.4 ^b^	80.1 ^ab^	83.2 ^a^	1.559	0.080
	1–28	58.6 ^ab^	53.8 ^b^	60.3 ^a^	62.0 ^a^	1.018	0.017
ADFI, g/(duck-d)	1–9	35.5	36.3	38.1	37.2	0.407	0.114
	9–14	77.2	74.4	80.4	80.9	1.219	0.209
	14–28	134.7	137.7	140.6	143.7	2.185	0.525
	1–28	90.2	92.2	94.9	95.8	1.227	0.364
F/G ratio	1–9	1.29	1.34	1.30	1.27	0.010	0.076
	9–14	1.30	1.39	1.33	1.34	0.017	0.420
	14–28	1.74 ^b^	1.91 ^a^	1.76 ^b^	1.73 ^b^	0.025	0.019
	1–28	1.54 ^b^	1.72 ^a^	1.58 ^b^	1.55 ^b^	0.023	0.006

BW, body weight. ADG, average daily gain. ADFI, average daily feed intake. F/G ratio, feed intake: weight gain. NC–negative group, basal diet without *E. coli* challenge. PC–positive group, basal diet with *E. coli* challenge. ANT–antibiotic group, basal diet + virginiamycin (30 mg/kg) with *E. coli* challenge. BS–basal diet + 2.5 × 10^9^ CFU/kg *B. subtilis* with *E. coli* challenge. ^a,b^ In the same row, values with different small letter superscripts mean significant difference (*p* < 0.05). Each replicate was considered as the experimental unit.

**Table 2:** Effect of *B. subtilis* on serum albumin, globulin, A/G ratio and LPS content of ducks challenged with *E. coli*.

Parameter	Days	Dietary Treatment				SEM	*p*-Value
		NC	PC	ANT	BS		
Albumin, (g/L)	9	10.18 ^a^	7.60 ^b^	8.25 ^ab^	9.30 ^ab^	0.351	0.034
	14	10.03 ^a^	7.50 ^b^	8.90 ^ab^	9.07 ^ab^	0.294	0.012
	28	9.02	8.27	9.37	8.45	0.426	0.808
Globulin, (g/L)	9	11.43 ^b^	15.08 ^a^	13.35 ^ab^	13.27 ^ab^	0.466	0.040
	14	10.83	13.06	11.35	11.00	0.827	0.164
	28	14.63	13.57	14.95	13.13	0.749	0.825
A/G ratio	9	0.86 ^a^	0.54 ^b^	0.61 ^b^	0.71 ^ab^	0.011	0.042
	14	0.89 ^a^	0.59 ^b^	0.77 ^ab^	0.81 ^a^	0.094	0.029
	28	0.66	0.60	0.63	0.65	0.009	0.073
LPS, (EU/mL)	9	0.56 ^b^	0.63 ^a^	0.60 ^a^	0.59 ^ab^	0.007	0.002
	14	0.58 ^c^	0.62 ^ab^	0.65 ^a^	0.61 ^bc^	0.008	0.002
	28	0.66	0.68	0.73	0.66	0.011	0.114

A/G ratio, albumin: globulin. NC–negative group, basal diet without *E. coli* challenge. PC–positive group, basal diet with *E. coli* challenge. ANT–antibiotic group, basal diet + virginiamycin (30 mg/kg) with *E. coli* challenge. BS–basal diet + 2.5 × 10^9^ CFU/kg *B. subtilis* with *E. coli* challenge. A/G ratio, albumin: globulin. ^a,b,c^ In the same row, values with different small letter superscripts mean significant difference (*p* < 0.05). The individual duck in each replicate was the experimental unit (*n* = 6).

**Table 3:** Characteristics of the reads from 12 duck’s jejunum libraries.

Samples ID	Clean Reads	Clean Bases	GC Content (%)	% ≥ Q30 (%)	Mapped Reads	Uniq Mapped Reads
PC-1	20,587,116	6,140,986,574	50.45%	95.07%	36,927,117(89.69%)	33,262,066(80.78%)
PC-2	26,608,953	7,945,804,612	51.41%	95.34%	47,310,598(88.90%)	42,009,221(78.94%)
PC-3	30,147,965	9,017,205,702	51.88%	94.67%	53,396,826(88.56%)	48,909,124(81.12%)
PC-4	31,570,825	9,433,393,492	51.96%	94.50%	57,606,737(91.23%)	53,044,012(84.01%)
PC-5	29,190,041	8,715,512,362	51.83%	94.60%	52,130,891(89.30%)	47,333,397(81.08%)
PC-6	30,843,988	9,213,607,330	51.72%	94.44%	54,666,734(88.62%)	48,497,682(78.62%)
BS-1	28,473,759	8,511,148,562	51.82%	94.94%	51,884,478(91.11%)	46,221,094(81.16%)
BS-2	29,369,238	8,772,354,542	51.87%	94.84%	53,531,602(91.14%)	49,849,931(84.87%)
BS-3	23,512,394	7,028,365,784	53.05%	94.78%	54,958,510(88.58%)	49,777,431(80.23%)
BS-4	27,698,720	8,275,074,754	51.89%	94.74%	48,687,386(87.89%)	42,982,959(77.59%)
BS-5	25,829,435	7,728,346,758	51.82%	94.32%	47,421,641(91.80%)	42,797,776(82.85%)
BS-6	25,960,910	7,758,803,536	52.15%	95.19%	45,702,452(88.02%)	40,362,236(77.74%)

Clean reads, counts of clean PE reads. Clean bases, total base number of clean data. GC content, percentage of GC in clean data. ≥Q30%, percentage of bases with Q-score no less than Q30. Mapped reads, counts of mapped reads and the proportion of that in clean data. Uniq mapped reads, counts of reads mapped to a unique position on reference genome and proportion of that in clean data. PC–positive group, basal diet with *E. coli* challenge. BS–basal diet + 2.5 × 10^9^ CFU/kg *B. subtilis* with *E. coli* challenge. 1~6 represent 6 replicates per treatment.

**Table 4:** The top 10 DEGs with known functions contained in ribosome pathway.

Gene ID	Gene Name	*p*-Value	Regulated	KEGG Pathway Annotation
101801662	RPL27A	<0.001	up	Ribosome (ko03010)
101792873	RPL32	<0.001	up	Ribosome (ko03010)
101803698	RPL22L1	<0.001	up	Ribosome (ko03010)
101794125	RPLP2	<0.001	up	Ribosome (ko03010)
101798421	RPL35A	<0.001	up	Ribosome (ko03010)
101793195	RPL8	<0.001	up	Ribosome (ko03010)
101793091	MRPS11	<0.001	up	Ribosome (ko03010)
101794248	MRPL23	<0.001	up	Ribosome (ko03010)
101798947	NHP2	<0.001	up	Ribosome biogenesis in eukaryotes (ko03008)
101804664	RPL30	<0.001	up	Ribosome (ko03010)

**Table 5:** The top 10 DEGs with known functions contained in oxidative phosphorylation pathway.

Gene ID	Gene Symbol	*p*-Value	Regulated	KEGG Pathway Annotation
101802630	NDUFV1	<0.001	up	Oxidative phosphorylation (ko00190)
101794884	NDUFB5	<0.001	up	Oxidative phosphorylation (ko00190)
101798419	NDUFA2	<0.001	up	Oxidative phosphorylation (ko00190)
101791901	NDUFA6	<0.001	up	Oxidative phosphorylation (ko00190)
101800107	NDUFB3	<0.001	up	Oxidative phosphorylation (ko00190)
101795772	COX5A	<0.001	up	Oxidative phosphorylation (ko00190)
119713461	COX5B	<0.001	up	Oxidative phosphorylation (ko00190)
101796089	COX7A	<0.001	up	Oxidative phosphorylation (ko00190)
101800937	COX7B	<0.001	up	Oxidative phosphorylation (ko00190)
101802805	ATP5MF	<0.001	up	Oxidative phosphorylation (ko00190)

## Data Availability

The original contributions presented in the study are included in the article/supplementary material, further inquiries can be directed to the corresponding author. The raw sequence reads of ducks were deposited in NCBI’s Sequence Read Archive (SRA) database and accessible through accession No. PRJNA835725.

## Supplementary Materials

The following supporting information can be downloaded at: https://www.mdpi.com/article/10.3390/antiox11101951/s1, Table S1: Analysis composition of basal diets and nutrient level (air-dry basis, %); Table S2: Primers used for the RT-qPCR in this study; Table S3: The KEGG pathway enrichment analysis of DEGs; Figure S1: The KEGG classification of DEGs.
