# Supplementary material for: Bacillus subtilis Protects the Ducks from Oxidative Stress Induced by Escherichia coli: Efficacy and Molecular Mechanism
Source: Antioxidants (Basel). 2022 Sep 29;11(10):1951. doi: 10.3390/antiox11101951 (PMC9598806; doi:10.3390/antiox11101951)
Supplement: Supplementary file 1 [file antioxidants-11-01951-s001.zip › antioxidants-1844923-supplementary.pdf]

Additional file 1:

Table S1. Analysis composition of basal diets and nutrient level (air-dry basis, %).

**Parameter**	**Content(1 to 14d)**	**Content(14 to 28d)**
Ingredient		
Corn	58.90	67.98
Soybean meal	32.43	22.78
Cottonseed meal	2.00	4.00
Soybean oil	2.28	1.10
Limestone	1.07	1.14
CaHPO4	1.71	1.49
NaCl	0.30	0.30
L-Lys	0.17	0.09
DL-Met	0.14	0.12
Vitamin premix[1)]	0.03	0.03
Microelement premix[2)]	0.10	0.10
Zeolite powder	0.87	0.87
Total	100.00	100.00
Nutrient level[3)]		
Metabolizable energy (ME) (Mcal/Kg)	2.90	2.90
CP	20.00	17.50
Total lysine	1.10	0.85
Methionine	0.45	0.40
Total lysine + Methionine	0.80	0.71
Ca	0.90	0.85
Total P	0.65	0.60
Available P	0.45	0.40

[1)] Amount provided per kilogram of diet: Vitamin A 10 000 IU, Vitamin D_3 2 000 IU, Vitamin E 10 IU, Vitamin K_3 2.5 mg, Vitamin B_1 1 mg, Vitamin B_2 6 mg, Vitamin B_3 10 mg, Vitamin B_5 40 mg, Vitamin B_6 3 mg, Vitamin B_{11} 0.3 mg, Vitamin B_{12} 0.01 mg, biotin 0.12 mg.

[2)] The mineral premix provided the following per kilogram of diets: Cu (as copper sulfate) 8 mg, Fe (as ferrous sulfate) 80 mg, Mn (as manganese sulfate) 60 mg, Zn (as zinc sulfate) 40 mg, Se (as sodium selenite) 0.15 mg, I (as potassium iodide) 0.35 mg.

[3)] Based on composition of ingredients provided by NY/T 2122-2012.

Additional file 2:

Table S2. Primers used for the RT-qPCR in this study.

**Genes**	**Accession number**	**Primer Sequence (5'-3') sense/antisense forward primer**	**Production length (bp)**
RPLP2	XM_038180742.1	Forward: GCTCCCGCAAAGATGCGTTA<br>Reverse: AGCTTGCCATTGCCCTGAG	197
MRPL23	XM_027458708.2	Forward: GCTCCCAGTACAGCAGAATGA<br>Reverse: GTGGTTCCTCTTGTTGTTCGC	122
NHP2	XM_038186634.1	Forward: ATTGTGCGGAAACTGACTGC<br>Reverse: AATGGCCTGACGAGCTTGATA	234
NDUFV1	XM_038179705.1	Forward: TTCCCTGCTGATGTGGGTGTTT<br>Reverse: TTGTTGACGTGGCCGGAGAT	167
COX7B	XM_027465058.2	Forward: CATGAGCCAAACTTCCATGACA<br>Reverse: GCCTGCGTGAACACATAACC	95
ATP5MF	XM_027468823.2	Forward: AGAGACACACCCAAGATGGC<br>Reverse: TACCTCTCATACCCTCTGCG	242
Novel-gene *β-actin*	EF667345.1	Forward: GCTATGTCGCCCTGGATTT<br>Reverse: GGATGCCACAGGACTCCATAC	160

Additional file 3:

Table S3. The KEGG pathway enrichment analysis of DEGs.

KEGG ID	Description	Gene ratio	Enrich factor	*P*-value	*Q*-value	Gene number	Trend
ko03010	Ribosome	13.83%	5.37	<0.001	<0.001	91	up
ko00190	Oxidative phosphorylation	6.99%	3.28	<0.001	<0.001	46	up&down
ko00860	Porphyrin and chlorophyll metabolism	1.52%	2.67	<0.001	0.123	10	up&down
ko04260	Cardiac muscle contraction	2.28%	1.76	0.019	0.763	15	up&down
ko04520	Adherens junction	2.74%	1.55	0.036	1.000	18	down
ko04623	Cytosolic DNA-sensing pathway	1.37%	1.87	0.043	1.000	9	up&down
ko00790	Folate biosynthesis	0.61%	2.5	0.065	1.000	4	up&down
ko03020	RNA polymerase	1.06%	1.75	0.095	1.000	7	up&down
ko00310	Lysine degradation	1.67%	1.53	0.097	1.000	11	up&down
ko04122	Sulfur relay system	0.46%	2.5	0.107	1.000	3	up
ko04144	Endocytosis	6.53%	1.2	0.109	1.000	43	up&down
ko00030	Pentose phosphate pathway	0.91%	1.73	0.123	1.000	6	up&down
ko04211	Longevity regulating pathway	0.15%	7.49	0.134	1.000	1	up
ko04341	Hedgehog signaling pathway - fly	0.15%	7.49	0.134	1.000	1	up
ko00920	Sulfur metabolism	0.46%	2.25	0.139	1.000	3	up&down
ko03050	Proteasome	1.22%	1.54	0.140	1.000	8	up
ko00120	Primary bile acid biosynthesis	0.61%	1.87	0.155	1.000	4	up
ko00240	Pyrimidine metabolism	2.74%	1.26	0.176	1.000	18	up&down
ko00330	Arginine and proline metabolism	1.37%	1.4	0.183	1.000	9	up&down
ko00630	Glyoxylate and dicarboxylate metabolism	0.91%	1.5	0.203	1.000	6	up&down

Gene ratio: The proportion of DEGs associated with pathway; Enrich factor: Ratio of gene numbers to all gene numbers annotated in the KEGG pathway; *Q*-value: *P*-value adjusted by method Benjamini and Hochberg; Gene number: The number of DEGs in each KEGG pathway; Trend: Expression alterations of genes associated with various pathways.

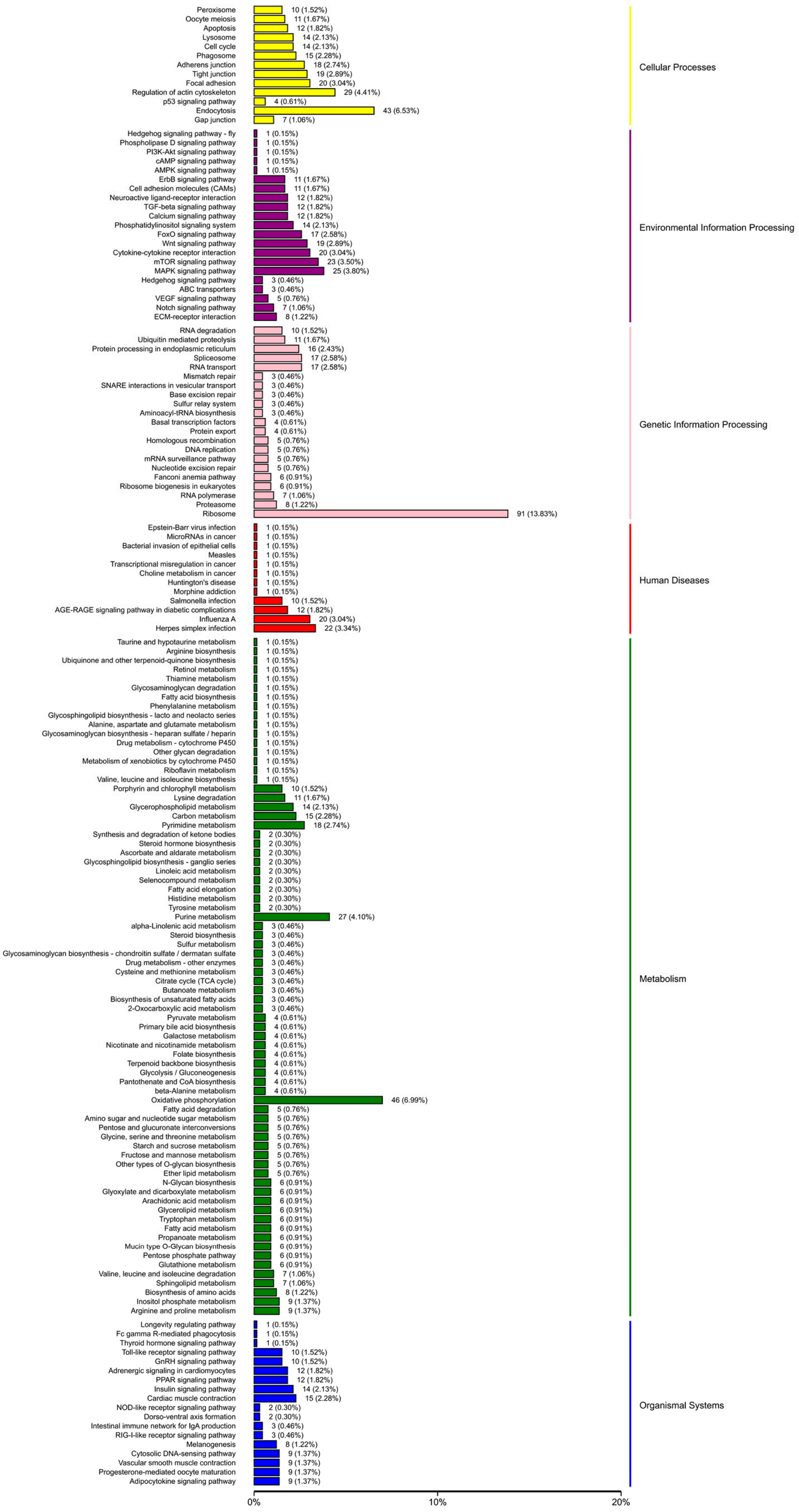
Peroxisome 10 (1.52%)
Oocyte meiosis 11 (1.67%)
Apoptosis 12 (1.82%)
Lysosome 14 (2.13%)
Cell cycle 14 (2.13%)
Phagosome 15 (2.28%)
Adherens junction 18 (2.74%)
Tight junction 19 (2.89%)
Focal adhesion 20 (3.04%)
Regulation of actin cytoskeleton 29 (4.41%)
p53 signaling pathway 4 (0.61%)
Endocytosis 43 (6.53%)
Gap junction 7 (1.06%)
Cellular Processes
Hedgehog signaling pathway - fly 1 (0.15%)
Phospholipase D signaling pathway 1 (0.15%)
PI3K-Akt signaling pathway 1 (0.15%)
cAMP signaling pathway 1 (0.15%)
AMPK signaling pathway 1 (0.15%)
ErbB signaling pathway 11 (1.67%)
Cell adhesion molecules (CAMs) 11 (1.67%)
Neuroactive ligand-receptor interaction 12 (1.82%)
TGF-beta signaling pathway 12 (1.82%)
Calcium signaling pathway 12 (1.82%)
Phosphatidylinositol signaling system 14 (2.13%)
FoxO signaling pathway 17 (2.58%)
Wnt signaling pathway 19 (2.89%)
Cytokine-cytokine receptor interaction 20 (3.04%)
mTOR signaling pathway 23 (3.50%)
MAPK signaling pathway 25 (3.80%)
Hedgehog signaling pathway 3 (0.46%)
ABC transporters 3 (0.46%)
VEGF signaling pathway 5 (0.76%)
Notch signaling pathway 7 (1.06%)
ECM-receptor interaction 8 (1.22%)
Environmental Information Processing
RNA degradation 10 (1.52%)
Ubiquitin mediated proteolysis 11 (1.67%)
Protein processing in endoplasmic reticulum 16 (2.43%)
Spliceosome 17 (2.58%)
RNA transport 17 (2.58%)
Mismatch repair 3 (0.46%)
SNARE interactions in vesicular transport 3 (0.46%)
Base excision repair 3 (0.46%)
Sulfur relay system 3 (0.46%)
Aminoacyl-tRNA biosynthesis 3 (0.46%)
Basal transcription factors 4 (0.61%)
Protein export 4 (0.61%)
Homologous recombination 5 (0.76%)
DNA replication 5 (0.76%)
mRNA surveillance pathway 5 (0.76%)
Nucleotide excision repair 5 (0.76%)
Fanconi anemia pathway 6 (0.91%)
Ribosome biogenesis in eukaryotes 6 (0.91%)
RNA polymerase 7 (1.06%)
Proteasome 8 (1.22%)
Ribosome 91 (13.83%)
Genetic Information Processing
Epstein-Barr virus infection 1 (0.15%)
MicroRNAs in cancer 1 (0.15%)
Bacterial invasion of epithelial cells 1 (0.15%)
Measles 1 (0.15%)
Transcriptional misregulation in cancer 1 (0.15%)
Choline metabolism in cancer 1 (0.15%)
Huntington's disease 1 (0.15%)
Morphine addiction 1 (0.15%)
Salmonella infection 10 (1.52%)
AGE-RAGE signaling pathway in diabetic complications 12 (1.82%)
Influenza A 20 (3.04%)
Herpes simplex infection 22 (3.34%)
Human Diseases
Taurine and hypotaurine metabolism 1 (0.15%)
Arginine biosynthesis 1 (0.15%)
Ubiquinone and other terpenoid-quinone biosynthesis 1 (0.15%)
Retinol metabolism 1 (0.15%)
Thiamine metabolism 1 (0.15%)
Glycosaminoglycan degradation 1 (0.15%)
Fatty acid biosynthesis 1 (0.15%)
Phenylalanine metabolism 1 (0.15%)
Glycosphingolipid biosynthesis - lacto and neolacto series 1 (0.15%)
Alanine, aspartate and glutamate metabolism 1 (0.15%)
Glycosaminoglycan biosynthesis - heparan sulfate / heparin 1 (0.15%)
Drug metabolism - cytochrome P450 1 (0.15%)
Other glycan degradation 1 (0.15%)
Metabolism of xenobiotics by cytochrome P450 1 (0.15%)
Riboflavin metabolism 1 (0.15%)
Valine, leucine and isoleucine biosynthesis 1 (0.15%)
Porphyrin and chlorophyll metabolism 10 (1.52%)
Lysine degradation 11 (1.67%)
Glycerophospholipid metabolism 14 (2.13%)
Carbon metabolism 15 (2.28%)
Pyrimidine metabolism 18 (2.74%)
Synthesis and degradation of ketone bodies 2 (0.30%)
Steroid hormone biosynthesis 2 (0.30%)
Ascorbate and aldarate metabolism 2 (0.30%)
Glycosphingolipid biosynthesis - ganglio series 2 (0.30%)
Linoleic acid metabolism 2 (0.30%)
Selenocompound metabolism 2 (0.30%)
Fatty acid elongation 2 (0.30%)
Histidine metabolism 2 (0.30%)
Tyrosine metabolism 2 (0.30%)
Purine metabolism 27 (4.10%)
alpha-Linolenic acid metabolism 3 (0.46%)
Steroid biosynthesis 3 (0.46%)
Sulfur metabolism 3 (0.46%)
Glycosaminoglycan biosynthesis - chondroitin sulfate / dermatan sulfate 3 (0.46%)
Drug metabolism - other enzymes 3 (0.46%)
Cysteine and methionine metabolism 3 (0.46%)
Citrate cycle (TCA cycle) 3 (0.46%)
Butanoate metabolism 3 (0.46%)
Biosynthesis of unsaturated fatty acids 3 (0.46%)
2-Oxocarboxylic acid metabolism 3 (0.46%)
Pyruvate metabolism 4 (0.61%)
Primary bile acid biosynthesis 4 (0.61%)
Galactose metabolism 4 (0.61%)
Nicotinate and nicotinamide metabolism 4 (0.61%)
Folate biosynthesis 4 (0.61%)
Terpenoid backbone biosynthesis 4 (0.61%)
Glycolysis / Gluconeogenesis 4 (0.61%)
Pantothenate and CoA biosynthesis 4 (0.61%)
beta-Alanine metabolism 4 (0.61%)
Oxidative phosphorylation 46 (6.99%)
Fatty acid degradation 5 (0.76%)
Amino sugar and nucleotide sugar metabolism 5 (0.76%)
Pentose and glucuronate interconversions 5 (0.76%)
Glycine, serine and threonine metabolism 5 (0.76%)
Starch and sucrose metabolism 5 (0.76%)
Fructose and mannose metabolism 5 (0.76%)
Other types of O-glycan biosynthesis 5 (0.76%)
Ether lipid metabolism 5 (0.76%)
N-Glycan biosynthesis 6 (0.91%)
Glyoxylate and dicarboxylate metabolism 6 (0.91%)
Arachidonic acid metabolism 6 (0.91%)
Glycerolipid metabolism 6 (0.91%)
Tryptophan metabolism 6 (0.91%)
Fatty acid metabolism 6 (0.91%)
Propanoate metabolism 6 (0.91%)
Mucin type O-Glycan biosynthesis 6 (0.91%)
Pentose phosphate pathway 6 (0.91%)
Glutathione metabolism 6 (0.91%)
Valine, leucine and isoleucine degradation 7 (1.06%)
Sphingolipid metabolism 7 (1.06%)
Biosynthesis of amino acids 8 (1.22%)
Inositol phosphate metabolism 9 (1.37%)
Arginine and proline metabolism 9 (1.37%)
Metabolism
Longevity regulating pathway 1 (0.15%)
Fc gamma R-mediated phagocytosis 1 (0.15%)
Thyroid hormone signaling pathway 1 (0.15%)
Toll-like receptor signaling pathway 10 (1.52%)
GnRH signaling pathway 10 (1.52%)
Adrenergic signaling in cardiomyocytes 12 (1.82%)
PPAR signaling pathway 12 (1.82%)
Insulin signaling pathway 14 (2.13%)
Cardiac muscle contraction 15 (2.28%)
NOD-like receptor signaling pathway 2 (0.30%)
Dorso-ventral axis formation 2 (0.30%)
Intestinal immune network for IgA production 3 (0.46%)
RIG-I-like receptor signaling pathway 3 (0.46%)
Melanogenesis 8 (1.22%)
Cytosolic DNA-sensing pathway 9 (1.37%)
Vascular smooth muscle contraction 9 (1.37%)
Progesterone-mediated oocyte maturation 9 (1.37%)
Adipocytokine signaling pathway 9 (1.37%)
Organismal Systems
0%
10%
20%

Figure S1: The KEGG classification of DEGs
